# Automated trajectory planning for laser interstitial thermal therapy in mesial temporal lobe epilepsy

**DOI:** 10.1111/epi.14034

**Published:** 2018-03-12

**Authors:** Vejay N. Vakharia, Rachel Sparks, Kuo Li, Aidan G. O'Keeffe, Anna Miserocchi, Andrew W. McEvoy, Michael R. Sperling, Ashwini Sharan, Sebastien Ourselin, John S. Duncan, Chengyuan Wu

**Affiliations:** ^1^ Department of Clinical and Experimental Epilepsy UCL Institute of Neurology National Hospital for Neurology and Neurosurgery London UK; ^2^ Epilepsy Society MRI Unit Chalfont St Peter UK; ^3^ Wellcome/EPSRC Centre for Interventional and Surgical Sciences University College London London UK; ^4^ The First Affiliated Hospital of Xi'an Jiaotong University Xi'an Shaanxi China; ^5^ Department of Statistical Science University College London London UK; ^6^ Department of Neurology, Vickie and Jack Farber Institute for Neuroscience Jefferson Comprehensive Epilepsy Center Thomas Jefferson University Philadelphia PA USA; ^7^ Division of Epilepsy and Neuromodulation Neurosurgery Vickie and Jack Farber Institute for Neuroscience Thomas Jefferson University Philadelphia PA USA

**Keywords:** computer‐assisted planning, EpiNav, laser ablation, laser interstitial thermal therapy, mesial temporal sclerosis

## Abstract

**Objective:**

Surgical resection of the mesial temporal structures brings seizure remission in 65% of individuals with drug‐resistant mesial temporal lobe epilepsy (MTLE). Laser interstitial thermal therapy (LiTT) is a novel therapy that may provide a minimally invasive means of ablating the mesial temporal structures with similar outcomes, while minimizing damage to the neocortex. Systematic trajectory planning helps ensure safety and optimal seizure freedom through adequate ablation of the amygdalohippocampal complex (AHC). Previous studies have highlighted the relationship between the residual unablated mesial hippocampal head and failure to achieve seizure freedom. We aim to implement computer‐assisted planning (CAP) to improve the ablation volume and safety of LiTT trajectories.

**Methods:**

Twenty‐five patients who had previously undergone LiTT for MTLE were studied retrospectively. The EpiNav platform was used to automatically generate an optimal ablation trajectory, which was compared with the previous manually planned and implemented trajectory. Expected ablation volumes and safety profiles of each trajectory were modeled. The implemented laser trajectory and achieved ablation of mesial temporal lobe structures were quantified and correlated with seizure outcome.

**Results:**

CAP automatically generated feasible trajectories with reduced overall risk metrics (*P* < .001) and intracerebral length (*P* = .007). There was a significant correlation between the actual and retrospective CAP‐anticipated ablation volumes, supporting a 15 mm diameter ablation zone model (*P* < .001). CAP trajectories would have provided significantly greater ablation of the amygdala (*P* = .0004) and AHC (*P* = .008), resulting in less residual unablated mesial hippocampal head (*P* = .001), and reduced ablation of the parahippocampal gyrus (*P* = .02).

**Significance:**

Compared to manually planned trajectories CAP provides a better safety profile, with potentially improved seizure‐free outcome and reduced neuropsychological deficits, following LiTT for MTLE.

**Key Points**

- Laser interstitial thermal therapy (or LiTT) is a novel treatment offering a minimally invasive alternative to open surgery for patients with drug‐resistant MTLE
- The success of LiTT is related to the laser trajectory, as this determines the ablation volume of the mesial temporal structures and safety profile of the procedure
- Computer‐assisted planning (or CAP) provides a potential means of automating ablation trajectories by optimizing a number of complex parameters
- This is first study utilizing CAP for LiTT; CAP provided feasible trajectories in all patients that would have resulted in improved ablation volumes
- CAP significantly improved the safety profile of the trajectory and reduced collateral damage to nearby structures important for neuropsychological outcome

## 1 INTRODUCTION

Numerous operative techniques have been described to treat mesial temporal lobe epilepsy (MTLE) including anterior temporal lobe resection (ATLR) and selective amygdalohippocampectomy (SAH). The most common form of ATLR, based on the technique described by Spencer et al,^1^ involves resection of the lateral neocortex, temporal pole, and amygdala prior to intraventricular resection of the hippocampal head and body to the level of the tectal plate. More selective approaches, including transsylvian,^2^ transcortical,^3^ and subtemporal^4^ SAH, have not given better seizure freedom rates or neuropsychological outcomes.^5^, ^6^, ^7^ As fear of the operation is cited as a major factor preventing patients from undergoing surgery; a less‐invasive means of ablation may be more acceptable to patients and potentially increase surgical uptake. Thermal ablation is a lesioning technique that has been used in neurosurgery for many years with variable success.^8^, ^9^, ^10^ The main limitation to earlier methods was the unpredictable nature of thermal lesioning and the lack of real‐time monitoring. The combination of magnetic resonance (MR) thermography techniques with laser technology has allowed precise intracerebral lesioning to be performed using laser interstitial thermal therapy (LiTT).^11^ The majority of the clinical experience surrounding LiTT in epilepsy uses the Visualase system (Medtronic Inc., Minneapolis, MN, USA). The extent of the thermal ablation volume is monitored in real‐time with MR thermography.^12^ A critical part to the process, both in terms of safety and efficacy, involves the planning of the laser trajectory because this determines ablation safety, location, and volume. Previous studies have not shown ablation volume to be a predictive factor for post‐LiTT outcome, but they have suggested anatomical height of the amygdala and volume of residual unablated mesial hippocampal head as important factors.^13^, ^14^, ^15^, ^16^, ^17^

Limiting collateral damage to the lateral temporal neocortex, parahippocampal gyrus (PHG), and subcortical white matter fiber tracts has been suggested to improve neuropsychological outcomes compared to ATLR.^18^ Our aim is to validate the use of computer‐assisted planning (CAP) to maximize ablation of the amygdalohippocampal complex (AHC) while improving the safety profile when compared to previously implemented manually planned laser trajectories.

## 2 METHODS

### 2.1 Patient inclusion

Twenty‐five patients with mesial temporal sclerosis (MTS) that had previously undergone selective laser amygdalohippocampectomy (SLAH) at the Comprehensive Epilepsy Center at Thomas Jefferson University between 2012 and 2016 were included in the study. Patients underwent manual trajectory planning and SLAH ablation using the Visualase system (Medtronic Inc.). All patients underwent a comprehensive presurgical evaluation and postoperative follow‐up. Hemispheric language dominance was determined by functional MR imaging (fMRI). Outcome was assessed based on a modified Engel scale in which we compared patients who were seizure‐free with or without auras for 1 year or more (class 1) compared to all other outcomes (class 2‐4).^19^

### 2.2 EpiNav

EpiNav (Centre for Medical Imaging Computing, University College London, London, UK) is a multimodal imaging platform that has been used previously to undertake multitrajectory automated stereo‐electroencephalography (SEEG) electrode planning that is optimized to maximize contact with gray matter and distance from segmented vasculature while reducing intracerebral trajectory length, drilling angle to the skull, and overall risk.^20^, ^21^, ^22^, ^23^ We have now further developed EpiNav to plan automated hippocampal laser trajectories.

#### 2.2.1 Model generation

**Figure 1:**
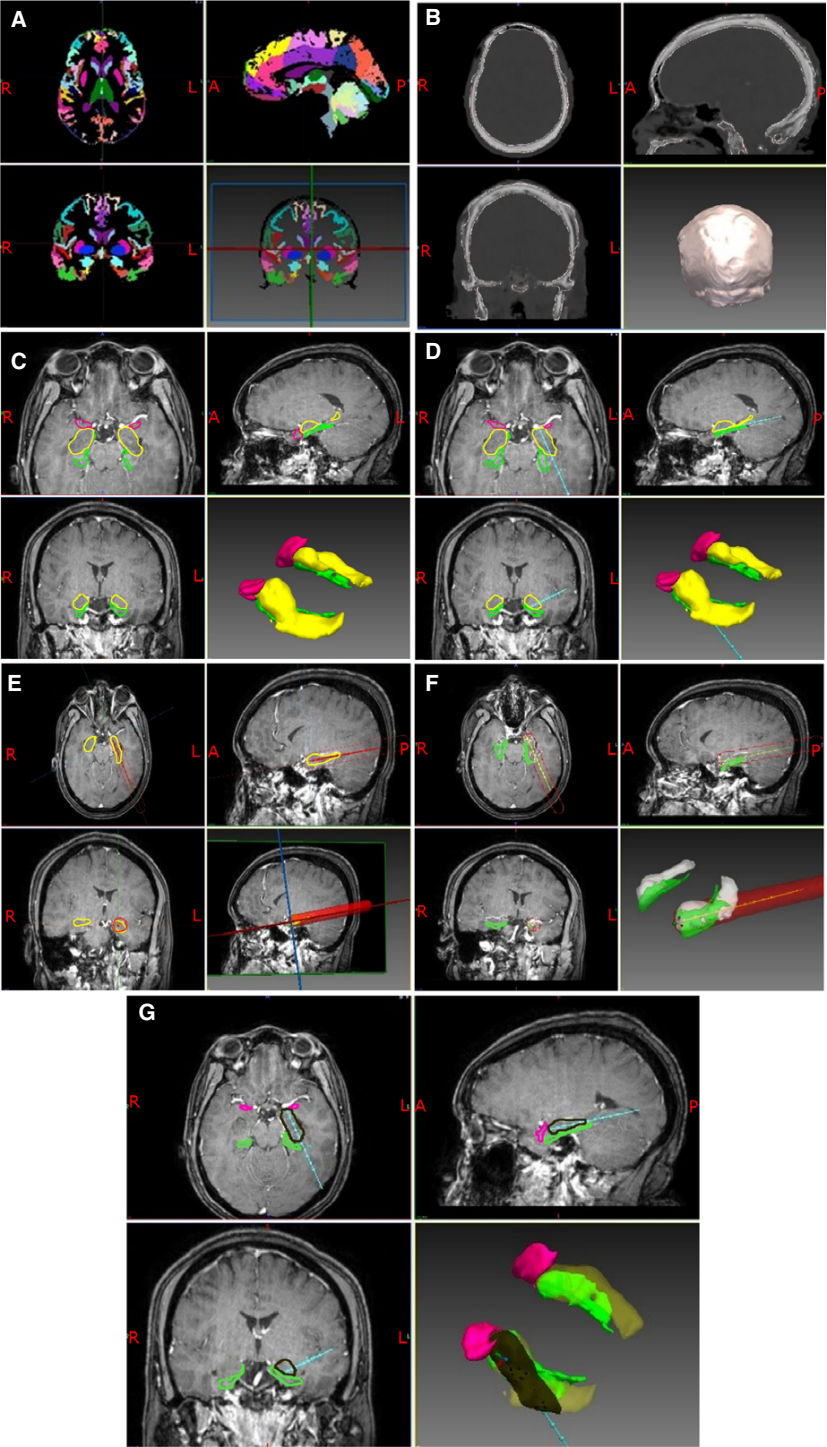
A, T1‐weighted MRI scans for each patient were used to generate geodesic information flow (GIF) brain parcellations. The whole brain is segmented into 140 separate anatomical structures that can be used to guide trajectory planning and model generation. B, Pseudo‐CT images were generated from the same T1‐weighted MRI scans to provide an image from which a model of the skull can be extracted. The external surface of the skull model is used to calculate the trajectory drilling angle, and the inner surface is used to calculate intracranial trajectory length. C, Models of the cortex, lateral ventricle, amygdala, hippocampus, entorhinal cortex, parahippocampal gyrus, gray matter ribbon, inferior occipital gyrus, middle occipital gyrus, inferior temporal gyrus, middle temporal gyrus, intracranial mask, and sulci are extracted from the GIF parcellation and combined with the skull model. In the image shown, the amygdalohippocampal complex is colored in yellow, entorhinal cortex in pink, and parahippocampal gyrus in green. The remaining models have been excluded for clarity. D, Based on the generated models the optimal trajectory is calculated to target the amygdala while preventing entry to the lateral ventricle, thereby maximizing contact with the hippocampus, distance from sulci and vasculature, and minimizing intracranial trajectory length and drilling angle to the skull. The calculated laser trajectory is shown in blue. E, A region of ablation is then modeled along the model laser trajectory. The Visualase system can ablate a diameter between 5 and 20 mm. A conservative maximum ablation diameter of 15 mm was applied to the model (red cylinder). F, Areas of overlap between the modeled laser ablation zone and the anatomical regions of interest were then extracted so that an estimation of the modeled ablation cavity could be calculated. The volume of each of the regions of interest within the modeled ablation cavity were calculated individually and as a whole. Amygdalohippocampal complex is shown in white and parahippocampal gyrus in green. G, Expected ablation cavity within the ROIs (black) showing the extent of mesial hippocampal head ablation. Amygdalohippocampal complex is shown in yellow, parahippocampal gyrus in green, and entorhinal cortex in pink

Utilizing a single, T1‐weighted MRI scan, a whole‐brain parcellation and pseudo–computed tomography (CT) images were generated using geodesic information flow (GIF) (Figure 1).^24^, ^25^ From the whole‐brain parcellation, anatomical regions of interest (ROIs) were extracted in an automated fashion including the lateral ventricles, hippocampus, amygdala, entorhinal cortex (ENCx), and parahippocampal gyrus (PHG). ROIs were then manually inspected to confirm anatomical accuracy. Potential trajectories were risk stratified based on cumulative distance from the sulci. The ventricular system was marked as an exclusion zone. Target regions for CAP were defined as the amygdala in which the central point was transformed by 3 mm medial, 3 mm anterior, and 3 mm inferior. The medial and inferior transformation were based on preliminary data to improve the ablation of the mesial hippocampal head and avoid heat transfer to the temporal stem and globus pallidus, respectively. The anterior transformation ensured that the trajectory target was situated on the anterior surface of the amygdala.^13^ The CAP algorithm selectively weights trajectories that maximize contact with the center of the AHC and provides a quantitative measure of this as a proportion of the entire structure. To facilitate cannulation of the long axis of the AHC, the entry point for CAP was assigned as the inferior occipital gyrus. Postablation MRI scans were assessed after generation of CAP trajectories in all patients. Manual trajectory planning was undertaken by the method described by Wu et al,^13^ utilizing the “posteroinferior corridor.” Here, an initial target point is placed in the center of the amygdala and a waypoint is placed between the occipital horn of the lateral ventricle and collateral sulcus. The trajectory is then extrapolated posteriorly to the cortical surface, with mediolateral adjustments to avoid vasculature, lateral ventricle, and sulci. Finally, the target point is extrapolated forward to the anterior surface of the amygdala.

#### 2.2.2 Safety metric calculation

Each of the CAP generated trajectories were reviewed by a neurosurgeon for feasibility. For both CAP and manually planned trajectories, the length, drilling angle to skull, minimum distance from critical structures, overall risk (cumulative distance from critical structures), and minimum distance of trajectory from brainstem were automatically calculated.^20^ Due to a lack of dedicated vascular imaging, it was not possible to segment vascular models for most cases in the study. To prevent potential conflict with cerebral vasculature, sulcal models were used as critical structures, as blood vessels are most likely to be present within sulci. Risk was measured as a cumulative distance from critical structures along the entire trajectory. Derivation of safety margins for stereotactic implantations was based on the sum of the diameter of the probe, average target inaccuracy, and 3 standard deviations of the inaccuracy.^26^ Average target accuracy was based on the method of implantation used.^27^ As a result, a 3 mm planning safety margin was applied. Distances from critical structures greater than 10 mm returned a risk of 0, whereas distances <3 mm returned a risk of 1.^21^ The overall risk for trajectory was calculated along the whole of the intracerebral length of the trajectory.

#### 2.2.3 Ablation‐zone modeling

Following generation of the CAP and manual trajectories for each patient “expected ablation zones” were produced using a 15 mm diameter dilation of the initial trajectory. ROIs within the expected ablation zone were extracted, and the paired structure volumes were calculated. For the purpose of anatomical volume, laser ablation zone modeling, and trajectory optimization, only the part of the hippocampus anterior to the tectal plate was considered.^1^ From the postablation MRI scans, the achieved ablation cavities were segmented manually. ROIs within the achieved ablation cavity were then extracted and compared with the calculated (expected) modeled ablation cavities of the manual trajectory to determine the validity of using a 15 mm diameter ablation‐zone estimation.

### 2.3 Statistical analysis

Statistical analysis was performed using SPSS 24. Mann‐Whitney‐*U* and Kruskal‐Wallis tests were performed for nonparametric comparisons. Correlation between ROI ablation volumes was calculated using a Pearson correlation. A *P* value < .05 was taken to be statistically significant.

### 2.4 Institutional review board approval

#15D.106 ‐ “Volumetric Analysis of MRIs in Patients with Mesial Temporal Lobe Epilepsy Treated with Stereotactic Laser Amygdalohippocampectomy.”

## 3 RESULTS

### 3.1 Patient demographics

Twenty‐five patients (12 male) with MTS who had previously undergone LiTT were studied. Post‐LiTT outcome data were available for all patients, with a mean follow‐up duration of 24.4 ± 14.1 months (mean ± standard deviation [SD]) (see Table 1). At last follow‐up, 44% (11/25) of patients were seizure‐free. One patient with a class 3 outcome underwent a further LiTT ablation 12 months following the first with no improvement in outcome. Three of the other patients with class 3 outcome and both patients with class 4 outcome subsequently underwent ATL lobe resection. ATL resulted in seizure freedom in 2 of the 3 patients with class 3 outcome.

**Table 1:** Summary of patient demographics

Patient	Age	Gender	Duration of epilepsy (y)	Hemispheric language dominance	Side of ablation	Follow‐up duration (mo)	Modified Engel outcome at last follow‐up	Complications following LiTT	Comments
1	25	Male	23	Left	Left	30	1	None	
2	35	Male	5	Left	Left	11	3	None	
3	29	Male	16	Left	Left	10.5	3	Transient blurry vision, single episode of psychosis requiring hospitalization	
4	29	Female	2	Left	Left	15	2	None	
5	11	Male	4	Left	Right	8	3	None	Underwent ATL 8 mo post‐LiTT
6	56	Female	40	Left	Left	61.5	1	None	
7	57	Female	44	Left	Left	14	3	None	
8	19	Male	3	Left	Right	35	1	None	
9	65	Male	10	Left	Left	8	4	None	Underwent ATL 8 mo post‐LiTT
10	48	Female	40	Left	Right	26.5	2	None	Also has parafalcine meningioma
11	41	Female	15	Left	Left	41	1	None	
12	20	Female	19	Left	Right	18	3	Transient anxiety with panic attacks	Underwent ATL 18 mo post‐LiTT
13	66	Male	31	Left	Left	17	1	None	
14	23	Female	22	Left	Left	29.5	3	Contralateral superior quadrantanopsia	Underwent reablation 12 mo post‐LiTT
15	66	Female	Unknown	Left	Left	27	3	None	“Epilepsy since childhood”
16	29	Male	24	Left	Left	31.5	1	Transient increased anxiety	
17	54	Female	58	Left	Right	45	1	None	
18	52	Female	14	Left	Left	6	4	None	Underwent ATL 6 mo post‐LiTT
19	58	Male	8	Left	Left	4.5	2	Committed suicide	Preexisting mood disorder
20	52	Female	13	Right	Right	29	1	Transient CN IV palsy	
21	59	Male	37	Left	Left	37	1	None	
22	14	Male	Unknown	Left	Left	24	2	None	“Epilepsy since childhood”
23	34	Female	33	Left	Left	27	1	None	
24	49	Male	24	Left	Left	15	3	None	Underwent ATL 15 mo post‐LiTT
25	43	Female	15	Left	Left	40	1	None	
Mean	41.4	M:F = 12:13	21.7	L:R = 24:1	L:R = 19:6	26.5 (median)			
SD	17.2		14.9						

### 3.2 Trajectory characteristics

The mean trajectory length was 8 mm less with CAP than with manual planning (*P* = .007). CAP trajectories also resulted in a significant reduction in the calculated overall risk score (*P* < .001). (see Table 2).

**Table 2:** Summary of qualitative safety metrics for manual and CAP‐generated trajectories

	Manual trajectory (mean ± SD)	CAP trajectory (mean ± SD)	*P* value
Length (mm)	90 ± 12	82 ± 6	.007^a^
Drilling angle (deg)	31.1 ± 7.8	32.3 ± 8.5	.47
Proportion of trajectory within center of AHC	0.50 ± 0.40	0.55 ± 0.20	.66
Overall risk	2.02 ± 0.64	0.96 ± 0.20	<.001^a^

Comparison of safety metrics between manual and CAP‐planned trajectories revealed a significantly shorter intracranial length (*P* = .007) and reduced overall risk score (*P* < .001) with CAP trajectories. There was no significant difference between the drilling angle to the skull or the proportion of the trajectory within the center of the AHC.

a Denotes statistical significance with *P* < .05.

With the variability in the individual anatomy of the lateral ventricles, depth of the collateral sulcus, and extent of sclerosis of the hippocampus, a feasible entry point through the lateral aspect of the inferior occipital gyrus was achieved by CAP in 72% (18/25) of cases. In all instances, the amygdala, in which the center‐point was transformed by 3 mm medial, 3 mm anterior, and 3 mm inferior, could to be used as the target point. The remaining entry points traversed the lateral aspect of the middle occipital gyrus in 20% of cases (5/25) and the posterior‐most aspect of the middle temporal gyrus in 8% of cases (2/25).

### 3.3 ROI model ablation volumes

Following the implementation of a 15 mm laser ablation diameter to both the CAP and manually planned trajectories, CAP trajectories significantly increased the modeled ablation volume of the AHC from 2748 ± 771 mm^3^ (mean ± SD) to 3282 ± 605 mm^3^ (mean ± SD), equating to an extra 11.34% of the total anatomical volume (*P* = .0075) (See Table 3). Amygdala ablation volumes increased by an extra 15.7% of the total anatomical volume (*P* = .0004). The residual (unablated) depth of the mesial hippocampal head reduced by 73% (*P* < .001). CAP‐planned trajectories resulted in an 11.3% decrease of the anatomical volume of PHG being ablated (*P* = .02) and reduction in the distance of the center of the trajectory from the brainstem by 1.85 mm (*P* = .0052).

**Table 3:** Comparison of expected ablations between manual and CAP‐generated trajectories for individual anatomical structures represented as absolute volumes (mm^3^) and as percentage of the anatomical volume at baseline

Structure	Anatomical volume (mm^3^) (mean ± SD)	Manual trajectory ROI volume ablated (mm^3^) (mean ± SD)	Manual trajectory % ROI ablated (mean ± SD)	CAP trajectory ROI volume ablated (mm^3^) (mean ± SD)	CAP trajectory % ROI ablated (mean ± SD)	*P* value
Amygdala	1648.19 ± 359.53	739.84 ± 372.29	45.80 ± 20.45	994.03 ± 318.77	61.16 ± 15.82	.0004^a^
Hippocampus	2987.22 ± 477.36	2003.28 ± 565.33	67.68 ± 17.55	2079.32 ± 488.46	70.18 ± 14.44	.6152
AHC	4792.43 ± 735.75	2748.30 ± 771.30	57.82 ± 15.05	3282.49 ± 604.62	69.16 ± 11.54	.0075^a^
ENCx	2318.75 ± 562.01	246.85 ± 271.41	11.35 ± 13.85	212.89 ± 270.77	8.87 ± 10.77	.7005
PHG	3023.94 ± 506.75	621.94 ± 495.06	20.77 ± 16.15	358.60 ± 258.02	12.56 ± 9.78	.0243^a^
Total	10135.12 ± 1395.68	3686.26 ± 959.25	36.73 ± 9.76	3932.00 ± 793.52	39.31 ± 8.73	.3116
Residual (unablated) depth of MHH (mm)	N/A	4.45 ± 1.58	N/A	1.19 ± 1.37	N/A	<.0001^a^
Distance of trajectory from brainstem (mm)	N/A	11.75 ± 2.81	N/A	9.90 ± 2.18	N/A	.0052^a^

AHC, amygdalohippocampal complex; ENCx, entorhinal cortex; MHH, mesial hippocampal head; PHG, parahippocampal gyrus.

a Denotes statistical significance with *P* < .05.

### 3.4 Actual and expected cavity volumes

#### 3.4.1 Comparison of actual total ablation cavity volume and ROI ablation

Ablation cavities from the postablation images of the manually planned trajectories were manually segmented for all patients and volumes calculated. A total implemented mean ablation cavity volume of 6675 ± 2470 mm^3^ was achieved, whereas total volume of gray matter within the ablation cavity was 3259 ± 1352 (mean ± SD). The mean proportion of the implemented ablation cavity containing gray matter was 49%; the remaining half of the ablation cavity was white matter.

#### 3.4.2 Correlation of expected (modeled) versus achieved (implemented) cavity volumes

The volumes of the achieved AHC ablations were compared with the expected AHC ablations when a 15 mm diameter ablation zone was applied to the manually planned and CAP trajectories. The estimated correlation coefficient was 0.64 with 95% confidence interval ([CI] 0.38‐0.89), suggesting a significant linear association (*R* ^2^ = 0.535, *P* < .001). Differences between actual and modeled ablation volumes when calculated using a cylindrical 15 mm ablation zone are shown in Table 4.

**Table 4:** Comparison of achieved and expected ablation volumes for manually planned and implemented trajectories

Structure	Achieved manual trajectory ROI ablation	Expected manual trajectory ROI ablation	Estimation error as proportion of anatomical volume (%)
Amygdala	741.92 ± 423.93	739.84 ± 372.29	−2.08
Hippocampus	1630.32 ± 580.90	2003.28 ± 565.33	+12.49
AHC	2510.54 ± 887.46	2748.30 ± 771.30	+4.96
ENCx	269.23 ± 368.17	246.85 ± 271.41	−0.97
PHG	478.87 ± 447.05	621.94 ± 495.06	+4.73
Total ROIs	3258.59 ± 1351.81	3686.26 ± 959.25	+4.22

Expected ablation volumes are those modeled using a 15‐mm‐diameter symmetrical ablation zone. Error for each structure is calculated as a proportion of the anatomical volume at baseline. AHC, amygdalohippocampal complex; ENCx, entorhinal cortex; PHG, parahippocampal gyrus; ROI, region of interest.

#### 3.4.3 Correlation with seizure freedom outcome

There was no significant difference between seizure‐free outcome and absolute total volume of ROI ablation (*P* = .73) or residual depth of the mesial hippocampal head (*P* = .43). A trend was found between seizure‐free outcome and the baseline anatomical volume of the amygdala, but this failed to reach significance (*P* = .08).

## 4 DISCUSSION

### 4.1 Application of computer‐assisted planning (CAP) in neurosurgery

CAP was first introduced to neurosurgery during the 1980s as a means of calculating frame‐based coordinates during stereotactic brain biopsies.^28^ Advances have included the addition of multimodal imaging,^29^, ^30^ three‐dimensional (3D) model generation,^31^ pathology segmentation atlas, and whole‐brain parcellation integration.^21^ The most recent advances in CAP has been in automated trajectory planning for deep brain stimulation and SEEG procedures. Through the implementation of constraints such as maximizing distance from blood vessels, avoidance of crossing sulcal boundaries, ensuring an orthogonal drilling angle to skull, minimizing intracerebral trajectory length, and optimizing gray matter sampling, algorithms can provide trajectories with improved safety metrics at a fraction of the planning time.^21^, ^22^ Blinded external validation studies of CAP‐generated electrodes have shown that they achieve feasibility ratings similar to manually planned trajectories and may even provide feasible trajectories when manually planned trajectories are deemed infeasible.^23^ Using the EpiNav software we have applied parameters to automate LiTT trajectories for the management of MTS to improve trajectory safety metrics and maximize ROI ablation volumes beyond that of manually planned trajectories in a fully automated fashion (see Figure 1 for pipeline).

### 4.2 Correlation of ROI ablation with seizure and neuropsychological outcomes

In contemporary series, seizure‐free outcomes following LiTT for MTS have varied between 54%^16^ and 80%.^13^ In the study by Wu et al, seizure freedom was achieved in 80%. All patients had MTS, whereas in the study that achieved 54%, only 7 of 13 had unilateral MTS. This highlights the need for careful patient selection. A later study by Kang et al^17^ reported longer‐term follow‐up on the same patient cohort as Wu et al. Seizure freedom fell to 60% at 2 years. In another series, 23 patients had at least 1‐year follow‐up, and 65% of patients had an Engel class 1 outcome.^14^ Jermakowicz et al^14^ also report lack of ablation of the mesial hippocampal head as being associated with poorer outcome. Lateral trajectories through the hippocampus and lack of MTS also showed a trend toward poorer outcome. There was no relation between the absolute ROI ablation volume and seizure freedom rates or neuropsychological outcomes. These findings correlate with the results of the current study, whereby there was also no relationship between postoperative seizure freedom rates and absolute total ROI ablation volumes. We report seizure freedom rates of 44% at a median follow‐up of 26.5 months. This is slightly lower than other studies in the published literature, but it likely reflects the normal variation and impact of individual outcomes on group level statistics in small case series. Nevertheless, this remains a minimally invasive alterative to open temporal lobe resection, and repeat LiTT or open surgery can still be performed if LiTT is unsuccessful.

### 4.3 Potential effect on neuropsychological outcomes

Drane et al^18^ compared patients undergoing SLAH with standard or tailored ATLR, and showed in the dominant hemisphere that SLAH resulted in significantly less postoperative decline in famous face and common noun naming. In the nondominant hemisphere, ATLR resulted in a significant comparative decline in famous face recognition only. Given that both methods involve lesioning of the amygdala and hippocampus, it can be inferred that collateral damage to the surrounding cortical and subcortical structures compromises neuropsychological function. The CAP‐generated trajectories resulted in a significant reduction in the expected PHG ablation compared to manually planned trajectories. Furthermore, entry through the inferior occipital gyrus spares the lateral temporal neocortex, temporal pole, and temporal stem. Prospective studies are required to determine whether this will lead to less postoperative neuropsychological morbidity.

### 4.4 Optimization of laser trajectories

Few studies have critically assessed implemented trajectories to improve AHC ablation volume. Our aim was to validate CAP trajectories, with regard to AHC ablation volume and safety metrics, when compared to manually planned trajectories. Wu et al^13^ compared trajectories and ablation volumes after implementation of a systematic method of manual trajectory planning. The method described is similar to the approach automated by the CAP trajectories. This resulted in an increase in the amygdala ablation from 42% to 66% and hippocampal ablation from 52% to 61%. In the current study, CAP‐generated trajectories were anatomically constrained, and cumulative distance from the sulci was maximized and used as a basis of risk stratification. Given these constraints, there was a small window between the collateral sulcus and the inferior surface of the occipital horn of the lateral ventricle through which trajectories could pass, which Wu et al^13^ originally described as the “posteroinferior corridor.” Due to the anatomical variation in the depth of the collateral sulcus and the size of the occipital horn of the lateral ventricle, a trajectory through the inferior occipital gyrus was feasible in only 72% (18/25) of cases. In the remaining cases, a more lateral and superior entry point was required through the posterior middle temporal and the lateral middle occipital gyri, respectively. Even with the application of the systematic method to increase ablation volume of manually planned trajectories, as described by Wu et al,^13^ the CAP trajectories provided an increased ablation of the AHC volume by 11.34% and reduced the depth of the mesial hippocampal head remnant to ~1 mm. The incidence of significant intracranial hemorrhage following LiTT cannot be accurately distinguished from the literature due to the low number of published reports. Nevertheless, given that there were no hemorrhages in this case series, this does not mean that the risk of hemorrhage is zero. As a result, we implemented a risk‐stratification method based on the cumulative distance from critical structures such as vasculature or sulci (in cases where vascular segmentation could not be performed). Based on data from SEEG studies, we model risk from 0 to 1 along the entire length of the trajectory.^20^ Any point along the planned trajectory where a critical structure is within 3 mm is attributed a risk of 1, whereas those greater than 10 mm are given a risk of 0. In this study, CAP trajectories halved the overall trajectory risk.

### 4.5 Significance and limitations

Here we provide the first automated CAP pipeline for optimizing laser trajectory planning utilizing a single T1‐weighted MRI image. This system is fully customizable to allow the user to anatomically constrain both entry and target points, stratify for ROI contact (central core of hippocampus), as well as defining critical structures to be avoided. To date, the only independent prognostic factor for seizure outcome following LiTT is the residual (unablated) hippocampal head,^14^ which CAP trajectories would reduce. Furthermore, the safety profile of the trajectory, as determined by the cumulative distance from the sulcal segmentation, is improved. The implication is that CAP trajectories may result in improved seizure freedom rates and improved safety profiles, although this remains to be proven through a prospective clinical trial. If future prospective studies are to be undertaken to determine if ROI‐ablation volume correlates with improved seizure freedom rates, we estimate that ~250 patients would need to be enrolled to detect an increase seizure freedom rate of 20% with a power of 90% at a significance level of *P* = .05. The current study is underpowered to statistically detect such a difference.

LiTT is likely to become more prevalent for the treatment of MTLE, as short‐term outcomes have been shown to be comparable to open surgical intervention. As such, as the number of institutions performing LiTT increase, each will undergo a learning curve. The increase in adoption will inevitably lead to variability in patient outcomes and complication rates, making initial comparisons to other modalities difficult. CAP may provide a solution whereby a uniform and objective means of generating laser trajectories overcomes the initial learning curve, potentially providing sustained and reliable outcomes. As newer evidence emerges and experience grows, the algorithm can be modified continuously to ensure that optimal trajectories are implemented uniformly.

The accuracy of ROI segmentation is based on the parcellation algorithm implemented within the model development stage. In this study we implemented GIF,^25^ a whole‐brain parcellation, instead of a dedicated hippocampal segmentation. This has the added benefit of including nearby anatomical ROIs, such as the PHG and ENCx, as well as allowing ventricular, sulcal, and cortical entry ROI model generation simultaneously, at the relative expense of hippocampal segmentation accuracy. GIF was derived from healthy controls. As such, when applied to populations with MTS it tends to overestimate the size of the hippocampus. All GIF segmentations were checked manually at the time of model generation, and the oversegmentation of the hippocampus was minor. Given that the same segmentations were used for both manual and CAP trajectory assessment, any error in the parcellation would effectively cancel out.

Due to the retrospective nature of this comparison study, it was not possible to prospectively control for baseline image quality. In addition, the patients did not undergo dedicated vascular imaging, such as MR venogram, so vascular segmentation was not possible. Sulcal models were used as proxy critical structures to avoid deep vasculature, whereas trajectories that conflicted with surface veins, based on gadolinium enhance T1 images, were considered not feasible and the next risk‐stratified trajectory was selected. Future prospective studies should include standardized structural and vascular imaging protocols. The patient cohort was derived from a single center and limited to 2 surgeons. Further studies should aim to be multicenter in nature to validate the algorithm against variability in practice.

Finally, the application of a 15 mm diameter ablation zone around the CAP and manual trajectories to provide an “expected” ablation cavity was not an exact estimation of the actual “achieved” ablation volumes. One reason for this is that the laser ablation zone in vivo is not cylindrical, as the lateral ventricles and basal cisterns act as heat sinks dissipating the thermal energy. These anatomical features result in a nonlinear ablation cavity that could not be easily modeled based on current clinical experience, due to patient variability. The intimate proximity of the hippocampus to the lateral ventricle and basal cisterns may explain why the expected cavity, based on a uniform ablation zone, disproportionately overestimated hippocampal ablation compared to the other ROIs. The estimated ablation cavity for both manual and CAP‐generated electrodes were calculated in the same fashion, to ensure uniformity during the comparison and account for any potential inaccuracy.

## 5 CONCLUSION

We present a novel, fully automated CAP system for the generation of LiTT trajectories to maximize mesial temporal ROI ablations, improve trajectory safety metrics, and maximize the ablation of mesial hippocampal head when compared to manually planned and implemented trajectories. CAP also significantly reduces collateral damage to nearby structures, such as the parahippocampal gyrus, which may reduce the cognitive effects of the procedure. We have also validated a 15 mm diameter ablation zone model as a predictor of ROI ablation volume. Prospective studies of CAP are needed to determine if this method is associated with improved seizure outcomes and reduced neuropsychological deficits.

## AUTHOR CONTRIBUTIONS

Vejay N Vakharia ‐ Concept, study design, data acquisition, data analysis, manuscript production; Rachel Sparks ‐ Study design, technical input, manuscript production; Kuo Li ‐ Study design, data acquisition; Aidan O'Keeffe ‐ Data analysis, critical review of manuscript; Anna Miserocchi ‐ Critical review of manuscript; Andrew W McEvoy ‐ Critical review of manuscript; Michael Sperling ‐ Study design, critical review of manuscript; Ashwini Sharan ‐ Data acquisition, critical review of manuscript; Sebastien Ourselin ‐ Technical input, study supervision, critical review of manuscript; John S Duncan ‐ Concept, study supervision, critical review of manuscript; Chengyuan Wu ‐ Study design, data acquisition, study supervision, critical review of manuscript.

## DISCLOSURE

None of the authors has any conflict of interest to disclose. We confirm that we have read the Journal's position on issues involved with ethical publication and affirm that this report is consistent with those guidelines.
